# Spray Droplet Characterization from a Single Nozzle by High Speed Image Analysis Using an In-Focus Droplet Criterion

**DOI:** 10.3390/s16020218

**Published:** 2016-02-06

**Authors:** Sofija Vulgarakis Minov, Frédéric Cointault, Jürgen Vangeyte, Jan G Pieters, David Nuyttens

**Affiliations:** 1 The Institute for Agricultural and Fisheries Research (ILVO), Technology and Food Science Unit, Agricultural Engineering. Burgemeester Van Gansberghelaan 115, bus 1, Merelbeke 9820, Belgium; sofija.vulgarakis@gmail.com (S.V.M.); jurgen.vangeyte@ilvo.vlaanderen.be (J.V.); 2 Agrosup Dijon, Joint Research Unit Agroecology—26 Bd Dr Petitjean, BP 87999, Dijon cedex 21079, France; frederic.cointault@agrosupdijon.fr; 3 Department of Biosystems Engineering, Faculty of Bioscience Engineering, Ghent University, Coupure links 653, Ghent 9000, Belgium; jan.pieters@ugent.be

**Keywords:** high speed image analysis, spray characterization, piezoelectric droplet generator, in-focus criterion

## Abstract

Accurate spray characterization helps to better understand the pesticide spray application process. The goal of this research was to present the proof of principle of a droplet size and velocity measuring technique for different types of hydraulic spray nozzles using a high speed backlight image acquisition and analysis system. As only part of the drops of an agricultural spray can be in focus at any given moment, an in-focus criterion based on the gray level gradient was proposed to decide whether a given droplet is in focus or not. In a first experiment, differently sized droplets were generated with a piezoelectric generator and studied to establish the relationship between size and in-focus characteristics. In a second experiment, it was demonstrated that droplet sizes and velocities from a real sprayer could be measured reliably in a non-intrusive way using the newly developed image acquisition set-up and image processing. Measured droplet sizes ranged from 24 μm to 543 μm, depending on the nozzle type and size. Droplet velocities ranged from around 0.5 m/s to 12 m/s. The droplet size and velocity results were compared and related well with the results obtained with a Phase Doppler Particle Analyzer (PDPA).

## 1. Introduction

In recent years, advances in plant protection have contributed considerably to increasing crop yields in a sustainable way. Easy to apply and rather inexpensive, pesticides have proven to be very efficient. However, when pesticides are applied to crops, some of the spray may not reach the target, but rather fall outside the intended spray area. This can cause serious economic and environmental problems.

Most pesticides are applied using agricultural sprayers. These sprayers use hydraulic nozzles which break the liquid into droplets with a wide range of droplet sizes and velocities and determine the spray pattern. Small droplets are prone to wind drift, while large droplets can run off from the target surface and deposit on the soil. Therefore, efforts are being undertaken to come to a more sustainable use of pesticides which is more and more regulated by international environmental and water laws. One of the main challenges is to reduce spray losses and maximize spray deposition on the target and the resulting biological efficiency of the active ingredient by improving the spray characteristics and the spray application process. Because the mechanisms of droplets leaving a hydraulic spray nozzle are very complex and difficult to quantify or model, there is a need for accurate experimental quantification techniques. Few studies are available on selection of nozzles to provide adequate spray deposition and coverage of the plant protection products [1,2,3].

In the past, various measuring techniques [4] have been employed in the research on spray and atomization to investigate spray characteristics including droplet sizes and velocities. However, there are few optical measurement techniques that are able to perform simultaneous non-intrusive measurements of droplet size and velocity.

Due to the development of modern technology such as powerful computers and lasers, quantitative optical non-imaging light scattering spray characterization techniques have been developed for non-intrusive spray characterization: Phase Doppler Particle Analyzers (PDPA) [5,6], laser diffraction analyzers, e.g., Malvern analyzers [7] and optical array probes [8]. Among them, the PDPA has widely been tested and its usefulness for spray characterization recognized. The major drawback of the PDPA is that it can only measure a spherical droplet, which is not always the case in practice. In addition, it is a point—measurement technique, so results may differ between researchers and giving information on overall spray structure is beyond the capability of this laser device.

On the other hand, the limitations of the non-imaging techniques and the recent improvements in digital image processing, sensitivity of imaging systems and cost reductions have increased the interest in high speed imaging techniques for agricultural applications [9] in general and pesticide applications [10] in particular.

Imaging analyzers are spatial sampling techniques consisting of a light source, a camera and a computer with image acquisition and processing software. The small droplet sizes and high velocities of the ejected spray droplets make it a challenge to use imaging techniques for spray characterization. Imaging techniques based on the interferometric technique (Interferometric Particle Imaging—IPI—Global Sizing Velocimetry—GSV—and dual-beam light scatter interferometry) use light scattering and interference for a 2D spray characterization and the droplet measurements are derived from out of focus images [11,12,13].

In contrast, most other imaging techniques use backlight for the illumination of the droplets to acquire their shadowgraphs, from which droplet characteristics are extracted. They allow one plane at a time to be imaged with exposure times down to a microsecond [14]. The choice between the IPI and backlight imaging is driven by the spray’s density and droplet size. IPI is limited to a low spray density, measures only spherical droplets and can suffer from beam-steering effects [15].

**Figure 1:**
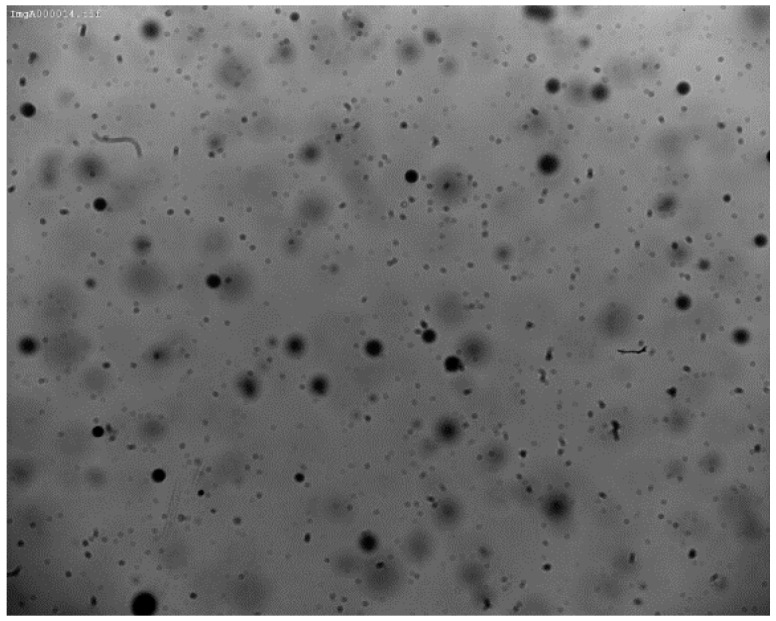
Example of a spray image with the different focus of the droplets.

Standard imaging techniques have however some limitations such as the difficulty of always having the same conditions for the acquisition for instance, and the need of optic and chromatic calibration and background identification. Moreover their data-acquisition rates are generally lower than those of the laser-based techniques. In particular, the use of backlight can impose limitations to the measurement accuracy which is related to the depth-of-field (DOF) effect. As droplets are scattered in the spray, not all droplet images are in-focus. DOF is the region in which the droplets are “acceptably” sharp or “in focus” and can thus be measured accurately. An in-focus droplet criterion is thus needed to select and further analyze these “in focus” droplets (Figure 1).

Chigier [16] indicated two possible sources of measurement errors caused by the DOF, *i.e.*, the ambiguity in defining the edge of an individual droplet when the droplet is located at some distance from the focal plane but still in the range of the DOF, and the dependence of the DOF on the droplet size itself.

In the literature, there are two major categories for in-focus droplet identification: the first one uses the gray level gradient at the droplet boundaries [10,17] while the second uses the contrast value between the droplet and the image background based on point spread function (PSF) [18,19]. The gray level gradient techniques provide information on the relation between droplet size and DOF. Kashdan *et al.* [17] used the thickness of blurred “halo” area at the edge of the droplet to determine the degree of droplet defocus. The gray level gradient at the droplet edges in the study of Lecuona *et al.* [10] was found by means of Sobel masks. Large droplets have a higher image contrast and can thus be measured over a greater distance to the lens than small droplets. Kashdan *et al.* [17] and Lecuona *et al.* [10] observed a linear relation between DOF and droplet diameter. Nevertheless, as the droplet size becomes smaller, the DOF decreases drastically, and small droplets are more likely to be missed from the spray droplet distribution.

Once the in-focus criterion is established, the next step is to accurately determine the droplet boundary. This includes droplet identification by determination of the threshold gray level for boundary detection, and treatment of the non-spherical and overlapped droplets.

This paper presents the proof of principle of a technique based on image processing for measuring the droplet size and velocity characteristics of agricultural hydraulic spray nozzles using an image acquisition system developed by Vulgarakis Minov *et al.* [20,21]. Firstly, an in-focus droplet criterion based on the gray level gradient was introduced to decide whether a droplet is considered to be in focus or not. A calibration system was devised using differently sized droplets generated with a piezoelectric droplet generator and glass nozzles in continuous mode, developed by Vulgarakis Minov *et al.* [21,22], at different distances from the focal plane and lens using a micro translation stage [10]. This enabled measurement of the gray level gradient and the in-focus parameter for every droplet size at various distances from the focal plane [10]. From here, a critical in-focus parameter (Inf_c_) was established for every droplet size and an in-focus droplet criterion was deduced to decide whether a droplet is in focus or not depending on its diameter and in-focus parameter. The focused droplet zone (FDZ) is defined in this study as the zone in which a droplet with a certain diameter is in focus.

Afterwards, the in-focus droplet criterion was applied to spray images of different hydraulic spray nozzles and the droplet characteristics were calculated. The effects of the nozzle type and nozzle size on spray droplet size and velocity characteristics were studied. Droplet size and velocity results were compared with an existing non-imaging droplet measuring technique, the PDPA [5].

## 2. Experimental Section

This section presents the image acquisition system and image analysis for setting up the in-focus droplet criterion.

### 2.1. Image Acquisition System and Measuring Set-Up

**Figure 2:**
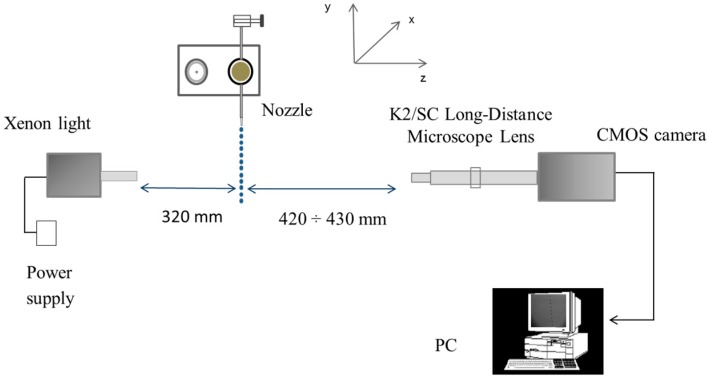
Image acquisition system for establishing the in-focus droplet criterion [21,22].

The image acquisition system for the development of the in-focus droplet criterion is shown in Figure 2 and is described in detail by Vulgarakis Minov *et al.* [20,21]. The system consists of a powerful xenon light (WOLF 5132, Knittlingen, Germany, 300 W) used as a background illumination against the droplet generator (Université de Liège, Gembloux, Agro-Bio-Tech, Walloon Region, Belgium) combined with an N3 HS (high speed) camera (IDT, Lommel, Belgium) with a 6 μs exposure time, a K2/SC Long-Distance Microscope System Lens (Infinity, Boulder, CO, USA) and a frame capture device Motion studio (IDT, Lommel, Belgium).

The piezoelectric droplet generator was positioned 320 mm from the xenon illumination and at a distance ranging between 420 and 430 mm from the lens. The camera, lens and illumination were aligned horizontally. A precision linear micro translation stage (0–25 mm, Edmund Optics, Barrington, NJ, USA) with a straight line accuracy of 10 μm moveable in the Z direction was attached to the lens. The droplet generator was implemented in continuous mode [21,22] using glass nozzles with orifice sizes of 261, 123, 67, 50 and 40 μm. These nozzle orifice sizes were chosen in order to produce a range of droplet sizes from around 100 μm up to 500 μm which is typical of most agricultural hydraulic spray nozzles. In the continuous mode, to produce a continuous stream of uniformly sized droplets, a suitable amplitude and frequency must be applied. The applied settings of the droplet generator (amplitude and frequency) and the corresponding actual droplet sizes at the focal plane are given in Table 1 [21,22].

**Table 1:** Actual droplet diameters in continuous mode for the different nozzle orifice sizes and continuous mode settings.

Nozzle Orifice Size (μm)	Settings in Continuous Mode A (V)/*f* (kHz)	Actual Droplet Diameter (μm) ± Standard Deviation
40	5.0/8.0	119.3 ± 2.6
50	5.0/8.0	164.6 ± 1.9
65	2.0/8.0	192.6 ± 1.3
65	5.0/8.0	222.9 ± 1.6
123	5.0/8.0	384.3 ± 0.8
261	5.0/8.0	489.7 ± 1.9
261	7.0/8.0	497.1 ± 2.0

A: Amplitude, *f*: frequency.

Multiple images of a Halcon ceramic calibration plate (2.5 mm × 2.5 mm) were taken and processed with HDevelop software to determine the accuracy of the optical system and possible optical errors as described in detail by Vulgarakis Minov *et al.* [21]. The setup resulted in a pixel size of 8.23 μm which made it possible to measure small droplets accurately and no image distortion occurred. The images were taken in full resolution (1280 × 1024 pixels) with a field of view (FOV) of 10.5 mm × 8.4 mm at a frequency of 1000 fps.

### 2.2. Image Acquisition for Setting Up the In-Focus Droplet Criterion

For establishing the in-focus droplet criterion, images were taken at different distances from the focal plane using all nozzles and settings given in Table 1. This was done by moving the translation stage (lens) towards and away from the focal plane in the range between 420 and 430 mm in steps of 50 μm (Figure 2). Thus, in this manner sequences of 200 “out-in-out of focus” images were taken with every nozzle/setting combination (Table 1).

**Figure 3:**
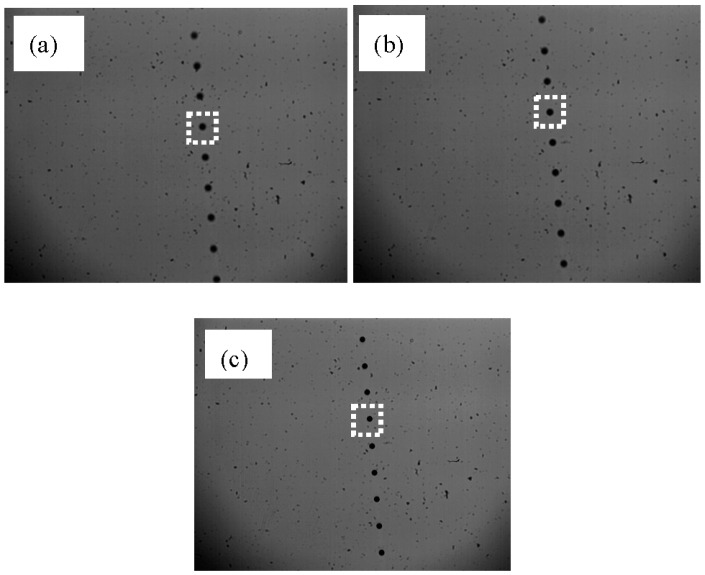
Droplet images in continuous mode using a nozzle with 65 μm orifice size at 5.0 V and 8.0 kHz at different distances from the lens: (**a**) 420 mm; (**b**) 423 mm; (**c**) 426 mm.

An example of images in continuous mode acquired using the nozzle orifice size of 65 μm at 5.0 V and 8.0 kHz, generating 222.9 μm droplets, at three distances from the focal plane is shown in Figure 3.

**Figure 4:**
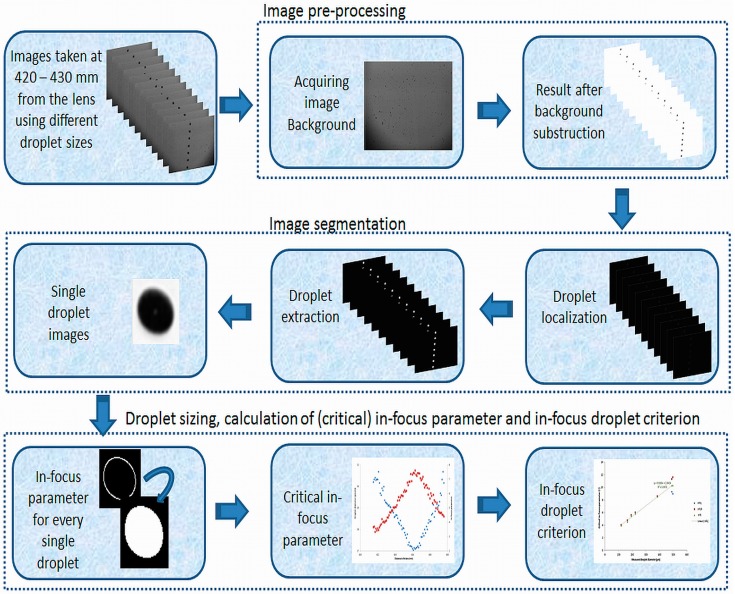
Flow chart of the image analysis algorithm for establishing the in-focus droplet criterion.

Once the images were acquired, a sequence of steps was employed to process and analyze them using Matlab and its image processing toolbox (Figure 4).

### 2.3. Image Analysis for Developing the In-Focus Droplet Criterion

The image analysis for setting up the in-focus criterion consisted of 3 steps: image pre-processing (Section 2.3.1), image segmentation (Section 2.3.2) and droplet sizing, calculation of (critical) in-focus parameter and in-focus droplet criterion (Section 2.3.3) (Figure 4).

#### 2.3.1. Image Pre-Processing

Image pre-processing aims at resolving problems due to lighting patterns or dirt on the lens, given that the light source can be non-homogeneous [23]. An image background was reconstructed from a set of 70 images with droplets. For each of these images, the background was selected based on its intensity histogram with a threshold value of 80% of the maximal pixel intensity of 255. These 70 background images were averaged and resulted in the final image background. Thus a background subtraction was performed on every single droplet image.

However, in general the image contrast was very low and the droplet boundaries were uncertain. Therefore, an illumination normalization was performed on the acquired images by rescaling the pixels such that exactly 1% of the pixels were saturated in order to maximize image contrast [24] and to facilitate the gradient characterization.

#### 2.3.2. Image Segmentation

Image segmentation was introduced to divide the image into sub-images of all individual droplets. The localization of droplets was performed by searching for sudden changes in the pixel intensity corresponding with the boundaries between a droplet and background. This was done by computing the intensity gradient on the whole image using the Sobel filter [24] (Figure 4). Further, the highlighted droplet contours were filled. Then, the image was binarized, *i.e.*, image pixels were distributed amongst two classes: droplets and background. The intensity threshold value was set at 85% of the maximum which was high enough to detect and maintain all droplets, even those out of focus. Because the droplets were not perfectly spherical, they were considered as ellipsoids. Their longest and shortest axes were measured to calculate the equivalent droplet diameter from the area [25].

Finally, sub-images of each detected droplet were constructed using the coordinates of the droplet center and the corresponding diameter. The size of the sub-images was equal to 1.5 times the droplet diameter, which was enough to capture the whole droplet and region of interest (Figure 4).

#### 2.3.3. Droplet Sizing, Calculation of (Critical) In-Focus Parameter and In-Focus Droplet Criterion

This step consisted of two main parts. The size of each detected droplet was calculated together with the corresponding in-focus parameter. In the second part, the critical in-focus parameter was calculated for each droplet size and the in-focus droplet criterion was established. The critical-in focus parameters and the resulting in-focus droplet criterion were used to select in-focus droplets.

(a) Droplet sizing and calculation of the in-focus parameters

**Figure 5:**
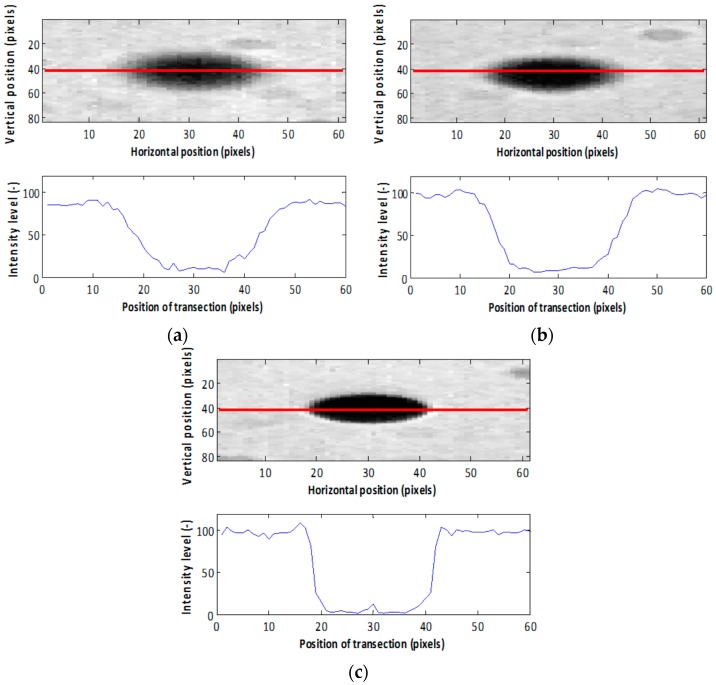
Detail and gray level intensity profiles from the marked droplets shown in Figure 3 at lens distances of, respectively, (**a**) 420 mm; (**b**) 423 mm and (**c**) 426 mm.

The droplet contours in the single droplet sub-images were extracted using a Canny edge detector [26] (Figure 4). When the contours were found, the droplet edge gradients, the gray level intensities of the droplet and background and the droplet size were calculated. However, the extracted droplets did not have the same gray level intensities and edge gradients because of their different positions relative to the focal plane (Figure 4). Figure 5a–c show three single droplet images (taken from Figure 3a–c) and their corresponding gray level intensity profiles across their centers. Ideally a droplet in focus has a flat intensity profile at the bottom close to 0 intensity level with steep edge gradients (Figure 5c). In this case, the droplet edges and size can be determined in an accurate way. In contrast, when a droplet is located at some distance from the focal plane and is out of focus (Figure 5a,b), there is an ambiguity in defining the droplet edges [17]. The gradients at the edges of the droplets reflect their degree of focus and can be used as a criterion based on which the droplets are chosen for measurement [27].

Therefore, the concept of in-focus parameter was introduced to select the in-focus droplets based on the gray level gradient, droplet diameter and gray level intensities of the background and droplet (Equation (1)) [10]:

(1) $$In-focus\;parameter\;=\;\frac{grad_{edge}}{I_{back}-I_{droplet}}*d$$

where *I_back_* (-) and *I_droplet_* (-) are image background and droplet gray level values, respectively, *d* is the droplet diameter (μm) and *grad_edge_* (-) is the gray level gradient at the droplet edge. Moreover, to eliminate the effect of light scattering inside every droplet, the *I_back_* (-) and *I_droplet_* (-) are calculated using a median filter. The in-focus parameter (-) was calculated for every detected droplet.

**Figure 6:**
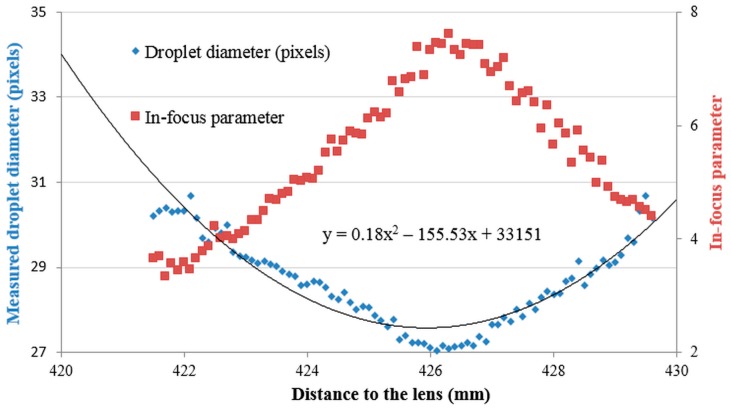
Measured droplet diameter and corresponding in-focus parameter for pictures taken of the 222.9 μm (27.1 pixels) droplet diameter at various distances from the lens.

As an example, Figure 6 presents the measured droplet diameters and the corresponding in-focus parameters for the experiments with a 222.9 μm (27.1 pixels) droplet at various distances from the lens. It can be seen that the in-focus parameter has a maximum at or near the position where the measured droplet diameter is minimal and corresponds with the actual droplet diameter. The further the distance from the focal plane the lower the in-focus parameter and the bigger the measured droplet diameter and the deviation with the actual droplet diameter. 

(b) Calculation of critical in-focus parameters and the in-focus droplet criterion

**Figure 7:**
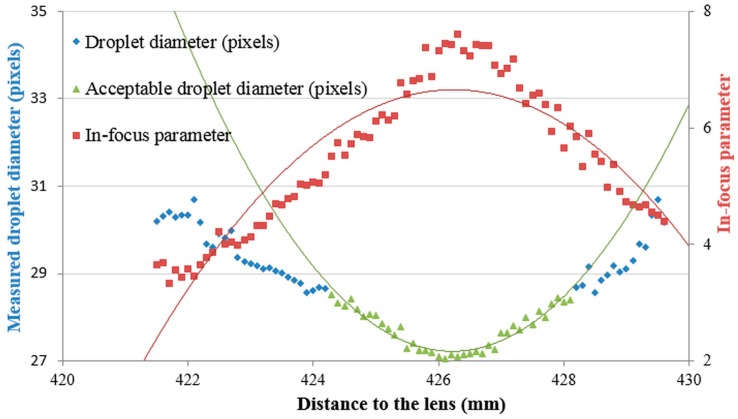
Measured droplet diameters and in-focus parameter values for pictures taken of the 222.9 μm droplet diameter at various distances from the lens.

To separate the droplets that are in-focus from the ones out of focus, a critical in-focus parameter (Inf_c_) was calculated for each of the seven droplet sizes (Table 1). The determination of Inf_c_ was done in several steps and is illustrated again for the 222.9 μm droplet size. Firstly, the minimal droplet diameter was estimated from the polynomial trend line of second order using all measured droplet diameters (27.6 pixels (227.1 μm), Figure 6). Then, an acceptable one pixel error value to this minimal droplet diameter was set corresponding with 28.6 pixels (235.3 μm) (Figure 7) meaning that we accept a deviation of up to 1 pixel between measured and actual droplet diameter. Hence, all droplets with a measured diameter below 28.6 pixels were considered to be in-focus, all others out of focus.

**Figure 8:**
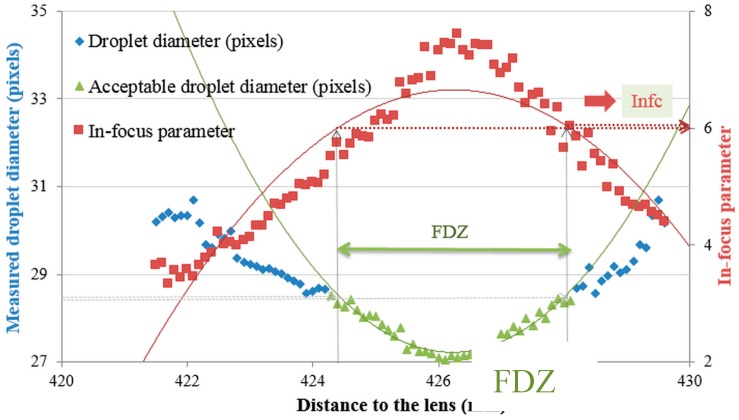
Critical in-focus parameters and FDZ for pictures taken of the 222.9 μm droplet diameter.

Next, another second order polynomial curve was fit only through these droplets considered in focus with an acceptable measured droplet diameter (Figure 8).

From this equation (y = 0.3953x^2^ − 336.95x + 71835) and the droplet diameter of 28.6 pixels, the corresponding distances to the lens were calculated (424.4 mm and 428.1 mm) (Figure 8). Combining these distances to the lens with the second order polynomial curve through the in-focus parameters, resulted into two values for the critical in-focus parameter, one on the left side, Inf_cL_ (6.0), and one on the right side, Inf_cR_ (6.1).The average of both values was considered the critical in-focus parameter Inf_c_ (6.0). All droplets with an in-focus parameter above Inf_c_ were considered in-focus. Besides, based on the distances to the lens at which the droplets were considered in focus, a focused droplet zone (FDZ) was defined. This is the zone around the focal plane in which droplets of a certain size are considered in-focus (Figure 8). For the 222.9 μm droplet size, the FDZ was 3.7 mm.

**Figure 9:**
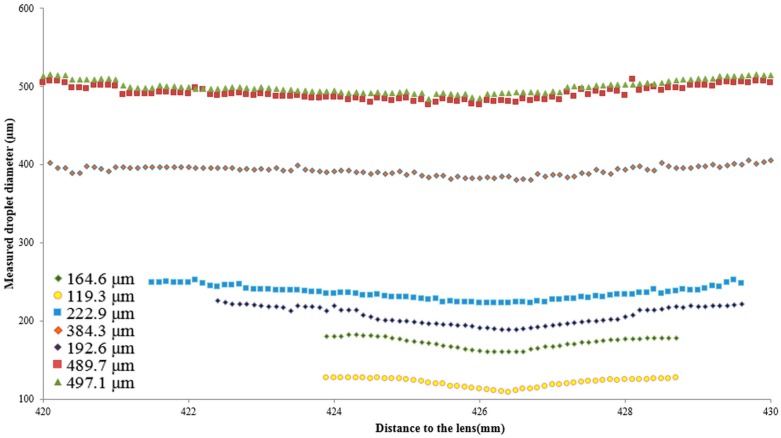
Relation between the measured droplet diameter and distance to the lens for droplet sizes ranging from 119.3 μm up to 497.1 μm.

**Figure 10:**
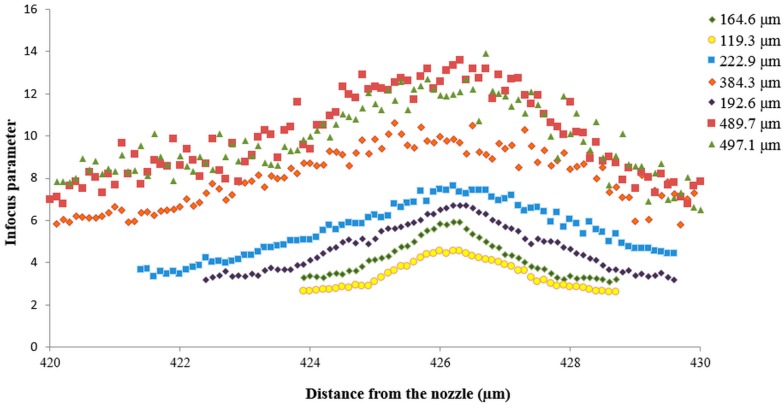
Relation between the in-focus parameter and distance to the lens for droplet sizes ranging from 119.3 μm up to 497.1 μm.

In order to evaluate the relations between Inf_c_, FDZ and droplet size, the procedure above was followed for all droplet sizes mentioned in Table 1. Results from these tests are shown in Figure 9 and Figure 10 and Table 2.

**Table 2:** Actual droplet diameters and their corresponding Inf_c_ and FDZ.

Actual Droplet Diameter (μm)	Inf_c_ (-)	FDZ (mm)
119.3	3.95	2.1
164.6	4.62	2.6
192.6	5.50	3.2
222.9	6.05	3.7
384.3	8.55	4.1
489.7	10.30	5.0
497.1	10.30	5.0

Measured droplet size was lowest at or near the focal plane (Figure 9). For each droplet size, the in-focus parameter was the biggest at or near the focal plane and mostly quickly dropped with increasing/decreasing distance to the focus plane [10,28] (Figure 9 and Figure 10). Besides, the smaller the droplet diameters, the narrower the corresponding curves in Figure 9 and Figure 10 meaning that smaller droplets completely disappeared closer to the focal plane than bigger droplets.

**Figure 11:**
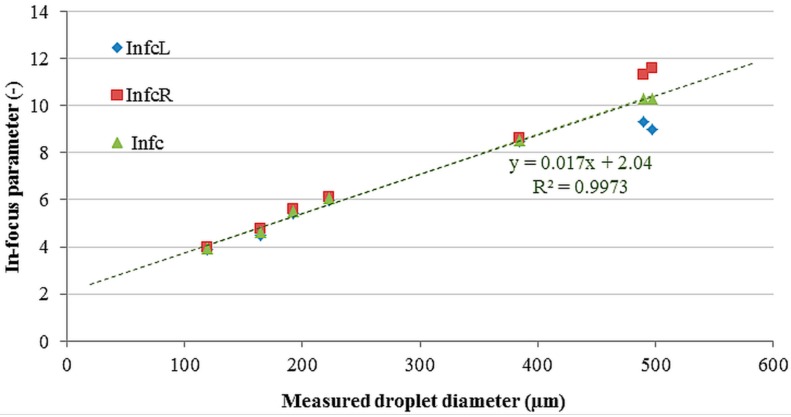
Relation between the critical in-focus parameter and measured droplet diameter.

Figure 11 and Table 2 show the relation between the critical in-focus parameter and the actual droplet diameter. It can be noted that the critical in-focus parameter increased almost linearly with the measured droplet diameter. In addition, this first order relation between droplet diameter (d) and Inf_c_ (Equation (2)) was used as the in-focus droplet criterion and used for selecting only the focused droplets in a real spray application.

(2) $$Inf_{c}\;=\;0.017\cdot d+2.04$$

**Figure 12:**
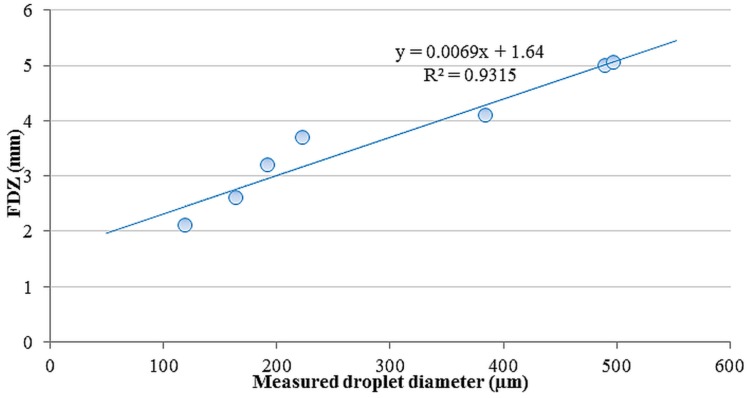
Relation between FDZ and measured droplet diameter.

Moreover, the FDZ increased linearly with measured droplet diameter (Figure 12). Droplets out of this zone were considered defocused and thus not measured.

## 3. Results and Discussion

### 3.1. Spray Droplet Characterization Using the In-Focus Droplet Criterion

**Figure 13:**
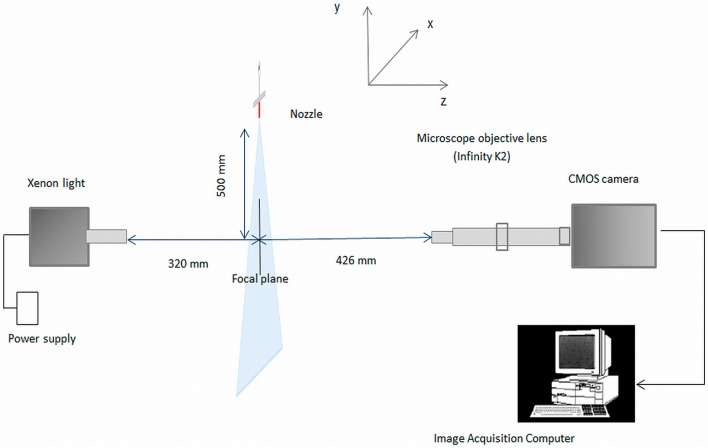
Image acquisition system for real spray droplet characterization.

A similar set-up as described for the in-focus droplet criterion was used for the real spray characterization using tap water as shown in Figure 13. Droplets dispersed in the spray were illuminated by a xenon light used as backlight. Spray droplet images were acquired by the HS CMOS camera.

**Figure 14:**
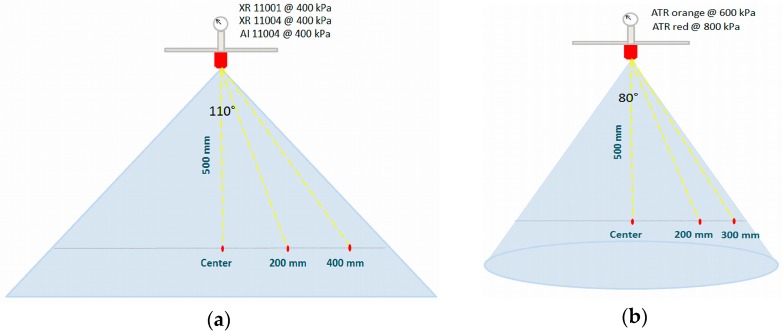
Spray measurement points for: (**a**) flat fan and (**b**) hollow cone nozzle.

In this study, five different hydraulic spray nozzles were selected: two hollow cone, two standard flat fan and one air inclusion flat fan nozzle. The selected nozzle-pressure combinations are presented in Table 3. The nozzle was always set between the lens and light source with the longest axis of the spray fan (in case of flat fan nozzles) parallel to the focal plane on an automated XYZ-transporter with a traverse range of 2.0 m by 2.2 m [5]. Images were acquired at 500 mm below the nozzle at three different positions: in the center, at 200 mm and at the edge of the spray (Figure 14). Based on the spray angles, the zone of the edge of the spray was defined at 400 mm for the flat fan nozzles and at 300 mm for the hollow cone nozzles. A schematic overview of the selected measurement points for every spray nozzle is given in Figure 14. For every nozzle and position combination, 500 images were taken at 1000 fps corresponding with a total time of 0.5 s.

The results obtained with the imaging system at every point were compared with the results measured at the same points with the PDPA. Measurement set-up, protocol and results have been described in detail by Nuyttens *et al.* [6].

**Table 3:** Manufacturer specifications of the tested hydraulic spray nozzles.

Nozzle Type	Nozzle	Pressure (kPa)	Spray Angle (°)	Nominal Flow Rate (L·min^−1^)
Hollow cone	Albuz ^a^ ATR orange	600	80	1.08
Hollow cone	Albuz ^a^ ATR red	800	80	1.73
Standard flat fan	TeeJet ^b^ XR 110 01	400	110	0.45
Standard flat fan	TeeJet ^b^ XR 110 04	400	110	1.82
Air inclusion flat fan	TeeJet ^b^ AI 110 04	400	110	1.82

^a^ Saint—Gobain Solcera, Evreux Cedex, France; ^b^ TeeJet Technologies, Wheaton, IL, USA.

Bigger droplets generally have a higher velocity than small droplets at 500 mm below the nozzle [5]. Therefore, small droplets remain longer in the FOV than large droplets. This means that one and the same droplet can be captured in several consecutive pictures and that the probability to measure a droplet more than once is bigger for smaller droplets than for bigger droplets. Therefore, not every consecutive image was analyzed but every ninth image resulting in a total number of 55 images for each nozzle at each position. This corresponds with a 9 ms time difference between analyzed images. This time difference was enough to ensure that one and the same droplet was not measured twice for the FOV of 10.5 mm × 8.4 mm and a minimal droplet velocity of 1 m/s [5].

**Figure 15:**
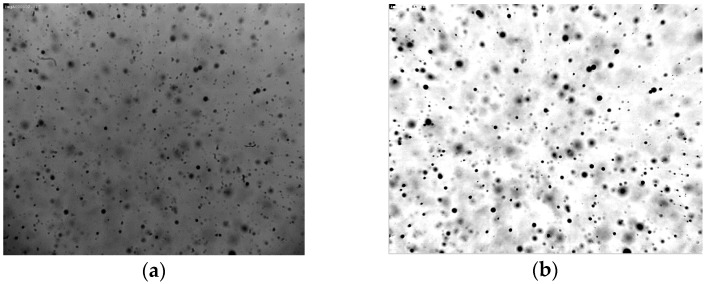
Example of spray droplet image with XR 110 04 nozzle at 400 kPa in the center: (**a**) before and (**b**) after pre-processing.

The image analysis for the selected images consisted of different steps: image pre-processing, image segmentation and droplet sizing and selection based on the in-focus criterion and droplet velocity calculation. Figure 15a shows an example of a typical spray image obtained with the XR110 04 nozzle in the center (Figure 14a). The image contains artefacts due to dust which have to be rejected, since they would represent a source of error.

**Figure 16:**
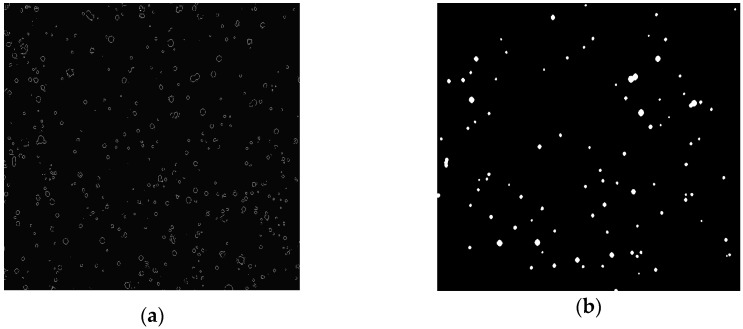
Spray droplet image shown in Figure 15b: (**a**) after droplet localization and (**b**) after applying morphological operations.

The image analysis was the same as for the droplet images using the piezoelectric generator in continuous mode described previously. The result is shown in Figure 15b. Every spray droplet has a different gray level and sharpness due to differences in the degree of focus and illumination (Figure 15b). Furthermore, blurred droplets can locally modify the background around droplets that are in-focus. Therefore, each droplet was separately analyzed by making sub-images. This image segmentation consisted of droplet localization and droplet extraction into single droplet sub-images. Droplet localization (Figure 16a) was achieved as described in Section 2.3. Each image was segmented into droplet and background regions by assigning pixels inside the droplet edge to the droplet and remaining pixels to the background [23,29]. Afterwards, the spray image was binarized for droplet detection. Morphological operations like opening, closing and filling the holes were applied (Figure 16b). Assuming the droplets are spherical, the next step included locating the center, calculating the area and estimating the equivalent diameter of every droplet. As in Section 2.3, single droplet images were extracted. In addition, spray droplets touching the image border were rejected for reasons of measurement accuracy.

**Figure 17:**
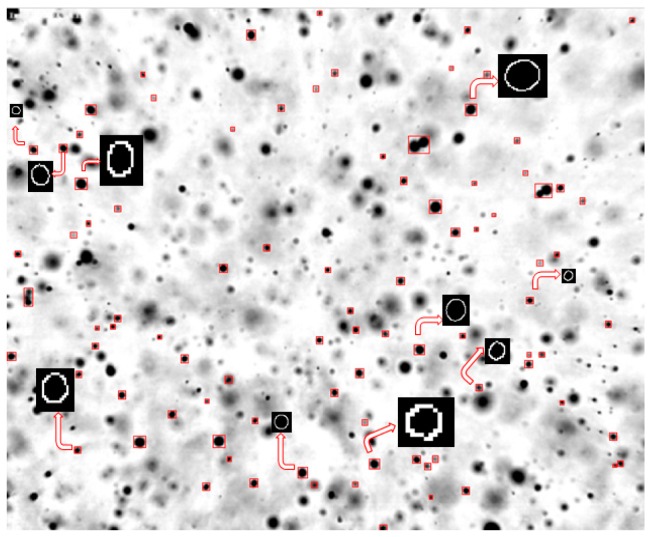
Examples of single droplet images after Canny edge detection.

Once the single droplet sub-images were extracted, a Canny edge detector was applied (Figure 17) [30]. The next step consisted of calculating the droplet edge gradients, the gray level intensities of droplet and background, and the droplet size by summing the pixels inside the droplet for every spray droplet, similarly as in Section 2.3.

In the final step the droplet in-focus criterion (Equation (2)) was applied to every spray droplet that satisfied the criterion to have a circularity bigger than 0.8. Overlapping droplets are not circular and therefore were not considered in the measurements.

Droplets having an in-focus parameter bigger than the corresponding Inf_c_ (based on the measured diameter and Equation (2)) were considered in focus and included in the spray droplet distribution results. All other droplets were rejected and not further used in the analysis.

Once the droplet center and position were determined, the next step involved droplet tracking to find the same droplet in two consecutive images, as well as the displacement vector and velocity. This was possible because of the large acquisition rate of the HS camera.

**Figure 18:**
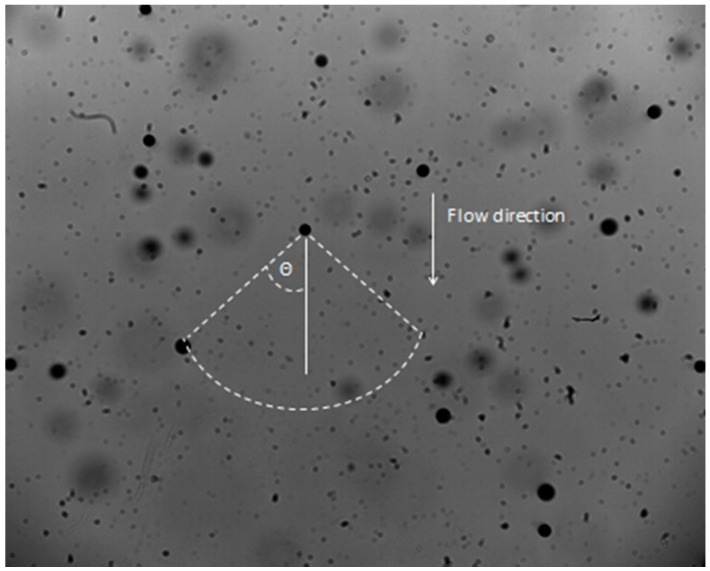
Droplet tracking 5 in the center of the spray.

However, few conditions related to the droplet diameter, droplet displacement and droplet velocity in a real spray application exist and are necessary in order to identify the same droplet on two successive images [22,31]. First is the condition of conservation of droplet diameter *i.e.*, the diameter of the candidate droplet on the consecutive images should not differ more than 2 pixels (16.5 μm) from the droplet on the first image [31].The second condition is related to the expected droplet direction based on the direction of the flow (Figure 18). A circular sector with an angle of confidence θ of ± 40° around the general flow direction was considered to define the search area to find the same droplet in the consecutive images. If the area is too big, this will result into droplet mismatches and velocity errors. On the other hand, if this area is too strictly defined, it will limit the detection of the fast droplets.

### 3.2. Spray Quality Parameters

The term “spray quality” is primarily used for describing the droplet size spectra of (agricultural) sprays. The spray characteristics like droplet size, velocity and direction influence the penetration and deposition of droplets. The efficiency of pesticide distribution often depends on droplet size [32] but hydraulic spray nozzles are not able to produce uniform droplets. A high coverage of the target is usually best achieved with small droplets [33] which are more subject to wind drift [34].

On the other hand, large droplets increase the risk of run off from target surfaces but have a higher kinetic energy which improves canopy penetration. The droplet size distribution is not homogeneous and depends on the position within the spray [35].

A comparison of the percentile number fractions produced by a nozzle to that of specific standardized reference nozzles classifies a droplet size spectrum. Number median diameter (NMD) is the droplet diameter for which 50% of the number of droplets is smaller than this value. Two nozzle-pressure combinations with the same NMD may actually produce a quite different droplet spectrum. Other important droplet characteristics are droplet velocity and direction (trajectory). NMV (number median velocity) is the droplet velocity for which 50% of the number of droplets is smaller than this value.

### 3.3. Spray Droplet Size Distribution

**Figure 19:**
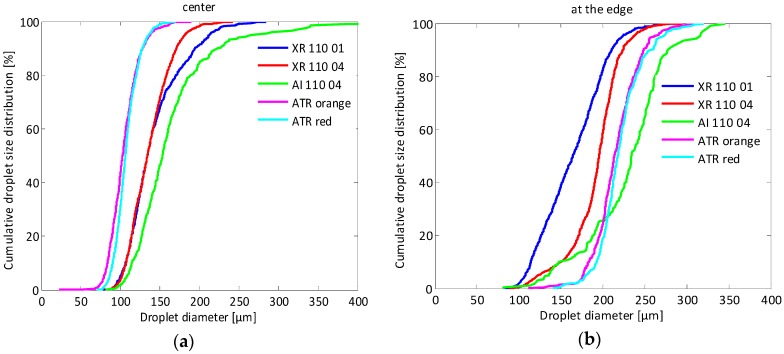
Cumulative droplet size distributions for the five nozzle-pressure combinations at 500 mm below the nozzle (**a**) in the center and (**b**) at the edge of the spray.

Figure 19 presents the cumulative droplet size distributions for the five nozzle-pressure combinations (Table 3) at two different measurement points at 500 mm below the nozzle, *i.e.*, center (Figure 19a) and at the edge of the spray (Figure 19b).

In the center of the spray, finer droplet size spectra were found for the hollow cone nozzles (ATR orange and red) followed by the standard flat fan nozzles (XR 110 01 and 110 04) while the coarsest droplets were found for the air inclusion flat fan nozzle (AI 110 04) which confirms previous results from, among others, Nuyttens *et al.* [6,36]. The difference between the ATR orange at 600 kPa and the ATR Red at 800 kPa was limited which confirms the PDPA results published by Dekeyser *et al.* [37]. Similarly, no differences were found in measured droplet sizes between the XR 110 01 and the XR 110 04 nozzle at this position. Only at the edge of the spray, a clear difference in droplet size distribution was observed between XR 110 01 and XR 110 04. The XR 110 01 produced much more small droplets resulting in a finer and wider droplet size distribution.

### 3.4. Spray Droplet Velocity Distribution

**Figure 20:**
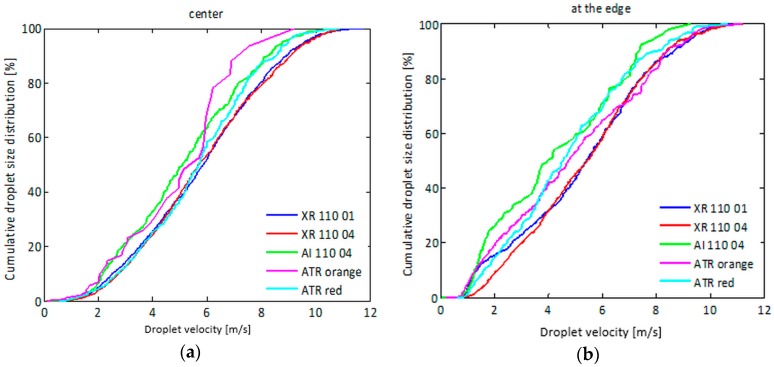
Cumulative droplet velocity distributions for the five nozzle-pressure combinations at 500 mm below the nozzle in the: (**a**) center and (**b**) edge of the spray.

Figure 20 presents the cumulative droplet velocity distributions for the five nozzle-pressure combinations (Table 3) for two different measurement points at 500 mm below the nozzle, *i.e.*, center (Figure 20a) and at the edge of the spray (Figure 20b).

In the center of the spray, no clear differences in droplet velocity distribution were observed for the different nozzles. The air inclusion nozzle tended to produce the slowest droplets at this position which was even more pronounced at the other positions. As a result, the steepest velocity distribution was measured for the air inclusion nozzle at all positions. Differences between nozzles were most pronounced at the edge of the spray with the slowest droplets for the air inclusion nozzle followed by both hollow cone nozzles. Highest velocities were here observed with both standard flat fan nozzles (XR 110 01 and XR 110 04).

### 3.5. Comparison between Imaging and PDPA Measuring Technique

**Figure 21:**
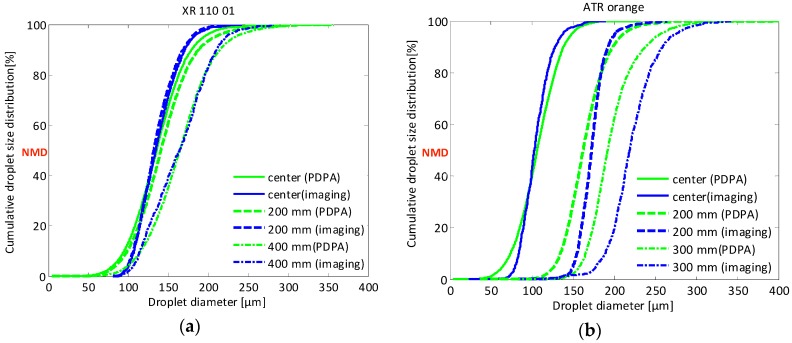
Cumulative droplet size distribution results using the imaging technique and PDPA for the: (**a**) XR 110 01 and (**b**) ATR orange at three measurement points.

In Figure 21, the cumulative droplet size distributions measured with two techniques, *i.e.*, imaging technique and PDPA at three different measurement points for standard flat fan nozzle XR 110 01 (Figure 21a) and hollow cone ATR orange nozzle (Figure 21b), are presented.

For the XR 110 01 nozzle (Figure 21a), a very good correlation between PDPA and imaging results was found at all measurement points. Only at the edge of the spray, the imaging technique resulted in a slightly coarser droplet distribution compared with the PDPA technique. The good correlation can be attributed to the small droplet size distribution of this nozzle and the lack of droplets >250–300 μm.

In addition, for the ATR orange nozzle (Figure 21b), a good correlation between PDPA and imaging results was found at the center although the imaging curves were a bit steeper for the reasons mentioned above.

In general, similar effects of nozzle type and measuring position were found with the PDPA technique as with the imaging technique although cumulative droplet size distributions curves gained with the imaging technique were steeper than those with the PDPA. This was caused by the fact that compared with the PDPA, the imaging technique generally measured a smaller number of small droplets and in some cases also a smaller number of the big droplets. Differences between both techniques can be attributed to the smaller amount of droplets measured with the imaging technique which increases the chance to miss one of the big droplets. In addition, no droplets below 24 μm were measured with the imaging technique while smaller droplets were measured with the PDPA.

**Figure 22:**
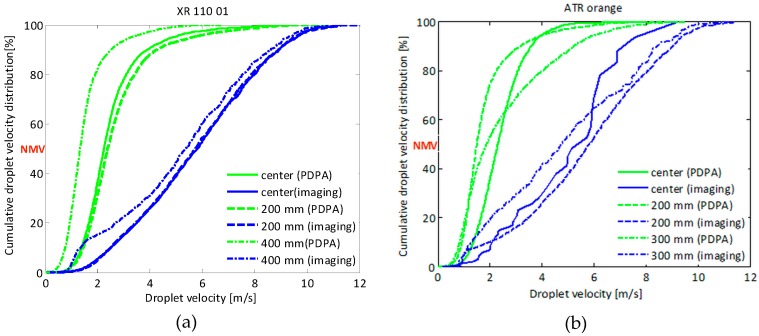
Cumulative droplet velocity distribution results using the imaging technique and PDPA for the: (**a**) XR 110 01 and (**b**) ATR orange at three measurement points.

In Figure 22, the cumulative droplet velocity distributions measured with the two techniques, at three different measurement points for standard flat fan nozzle XR110 01 (Figure 22a) and hollow cone ATR orange nozzle (Figure 22b) are presented. It can be observed that the droplet velocity distributions curves obtained with the imaging technique were shifted to higher values than the ones measured with the PDPA. This was most obvious with the standard flat fan XR 110 01 and the hollow cone ATR nozzle. This can partly be explained by the fact that the PDPA is only measuring droplet velocities in one dimension (vertically) and hence underestimates the actual droplet velocity. That is why differences between imaging and PDPA were generally most pronounced at the edge of the spray.

## 4. Conclusions

This paper presented the feasibility of using a technique based on image processing for measuring the droplet size and velocity characteristics of agricultural hydraulic spray nozzles, as a more versatile and cheaper alternative to existing methods such as PDPA.

Firstly, an in-focus droplet criterion based on the gray level gradient was introduced to decide whether a droplet is considered in focus or not. Differently sized droplets generated with a piezoelectric generator and glass nozzles in continuous mode at different distances to the focal plane and lens were measured. This enabled determination of the gray level gradient and in-focus parameter for every droplet size. From this, a critical in-focus parameter (Inf_c_) was established for every droplet size and an in-focus droplet criterion was deduced to decide whether a droplet is considered in focus or not depending on its diameter and in-focus parameter. In this study, the focused droplet zone (FDZ) was defined as the zone in which a droplet with a certain diameter is in focus and a linear relation between droplet size and FDZ was found.

Afterwards, the in-focus droplet criterion was applied to spray images of different hydraulic spray nozzles and the droplet size and velocity characteristics were calculated. The effects of the nozzle type, and nozzle size and measuring position on spray droplet characteristics were studied.

The droplet size and velocity results from the imaging technique have shown that it is possible to measure the spray characteristics in a non-intrusive way using image acquisition set-up and image processing. Measured droplet sizes ranged from 24 μm up to 543 μm depending on the nozzle type and size. Droplet velocities ranged from around 0.5 m/s up to 12 m/s. Similar effects of nozzle type and measuring position on droplet sizes as well as on droplet velocities were found with the imaging technique as with the PDPA or the droplet size and velocity, respectively.

The developed imaging technique can be seen as an alternative to the well-established PDPA. The droplet diameter and velocity characteristics showed satisfactory agreement with the results measured with the PDPA. When compared with the PDPA, the imaging technique generally measured less small droplets and in some cases also less big droplets. Differences between both techniques can be attributed to the fact that the smallest measured droplet size with the imaging system was 24 μm while smaller droplets were measured with the PDPA. In addition, the number droplets measured with the imaging technique was much smaller compared with the PDPA which increases the chance to miss one of the biggest droplets. This can be improved by taking more images. Differences in droplet velocity characteristics between both techniques can be attributed to the fact that the PDPA is only measuring droplet velocity in one dimension and hence underestimates the actual droplet velocity. In addition, the imaging technique applied did not allow the measurement of droplets faster than about 12 m/s based on the FOV and the acquisition rate while some droplets with higher speeds were observed with the PDPA mainly for the XR 110 04. The imaging system can be further improved to be able to measure at a higher frame rate with the same accuracy.

Although the approach proposed in this paper can be considered a new and innovative step to in-field spray droplet characterization and an alternative to expensive laser and holographic techniques, it is still a proof of principle. Several problems still need to be solved in order to be able to reliably measure droplet size and velocity distributions in dense sprays as encountered in real (agricultural) spray applications. Issues to be addressed include amongst others the improved separation of overlapping droplets and the characterization of the impact of the optical properties of the droplets on the results. In order to apply this method to sprayers in the field, the method and set-up also need to be modified to deal with moving nozzles, while real-time processing might require improvement of the proposed algorithms.

## Abbreviations

**Nomenclature Abbreviation**	**Description**
A	Amplitude
d	Droplet diameter
DOF	Depth-of-field
f	Frequency
FOV	Field of view
FDZ	Focused droplet zone
grad_edge_	Gray level gradient at the edge
HS	High speed
I_back_	Gray level of the image background
I_droplet_	Droplet gray level
Inf_c_	Critical in-focus parameter
Inf	In-focus parameter
NMD	Number median droplet
NMV	Number median velocity
PDPA	Phase Doppler Particle Analyzer
PSF	Point spread function

## References

[1] Azimi A.H., Carpenter T.G., Reichard D.L. (1985). Nozzle spray distribution for pesticide application. Trans. ASAE.

[2] Zhu H., Rowland D.L., Dorner R.C., Sorensen R.B. (2002). Influence of plant structure, orifice size and nozzle inclination on spray penetration into peanut canopy. Trans. ASAE.

[3] Foqué D., Nuyttens D. (2011). Effects of nozzle type and spray angle on spray deposition in ivy pot plants. Pest Manag. Sci..

[4] Rhodes M.J. (2008). Introduction to Particle Technology.

[5] Nuyttens D. (2007). Drift from Field Crop Sprayers: The Influence of Spray Application Technology Determined Using Indirect and Direct Drift Assessment Means. Ph.D. Thesis.

[6] Nuyttens D., De Schampheleire M., Verboven P., Brusselman E., Dekeyser D. (2009). Droplet Size and Velocity Characteristics of Agricultural Sprays. Trans. ASAE.

[7] Stainier C., Destain M.F., Schiffers B., Lebeau F. (2006). Droplet size spectra and drift effect of two phenmedipham formulations and four adjuvants mixtures. Crop Prot..

[8] Teske M.E., Thistle H.W., Hewitt A.J., Kirk I.W. (2002). Conversion of droplet size distributions from PMS Optical Array Probe to Malvern Laser Diffraction. At. Sprays.

[9] Hijazi B., Decourselle T., Vulgarakis Minov S., Nuyttens D., Cointault F., Pieters J.G., Vangeyte J., Constantin V. (2012). The Use of High-Speed Imaging System for Applications in Precision Agriculture. New Technologies: Trends, Innovations and Research.

[10] Lecuona A., Sosa P.A., Rodriguez P.A., Zequeira R.I. (2000). Volumetric characterization of dispersed two-phase flows by digital image analysis. Meas. Sci. Technol..

[11] Graßmann A., Peters F. (2004). Size measurement of very small spherical particles by Mie Scattering Imaging (MSI). Part. Part. Syst. Charact..

[12] Bachalo D.W. (1980). Dual beam light-scatter interferometry. Appl. Opt..

[13] Kannaiyan K., Sadr R. (2014). Effect of fuel properties on spray characteristics of alternative jet fuels using global sizing velocimetry. At. Sprays.

[14] Ju D., Shrimpton J.S., Hearn A. (2012). A Multi-Thresholding Algorithm for Sizing out of Focus Particles. Part. Part. Syst. Charact..

[15] Berg T., Deppe J., Michaelis D., Voges H., Wissel S. (2006). Comparison of Particle Size and Velocity Investigations in Sprays Carried out by Means of Different Measurement Techniques.

[16] Chigier N. (1991). Optical Imaging of Sprays. Prog. Energy Combust. Sci..

[17] Kashdan J.T., Shrimpton J.S., Whybrew A. (2007). A digital image analysis technique for quantitative characterisation of high-speed sprays. Opt. Lasers Eng..

[18] Kim K.S., Kim S.S. (1994). Drop sizing and depth-of-field correction in TV imaging. At. Sprays.

[19] Malot H., Blaisot J.B. (2000). Droplet size distribution and sphericity measurements of low-density sprays through image analysis. Part. Part. Syst. Charact..

[20] Vulgarakis Minov S., Cointault F., Vangeyte J., Pieters J.G., Nuyttens D. (2015). Development of High-Speed Image Acquisition Systems for Spray Characterization Based on Single-Droplet Experiments. Trans. ASABE.

[21] Vulgarakis Minov S. (2015). Integration of Imaging Techniques for the Quantitative Characterization of Pesticide Sprays. Ph.D. Thesis.

[22] Vulgarakis Minov S., Cointault F., Vangeyte J., Pieters J.G., Nuyttens D. (2015). Droplet generation and characterization using piezoelectric droplet generator and high speed imaging techniques. Crop Prot..

[23] Castanet G., Dunand P., Caballina O., Lemoine F. (2013). High-speed shadow imagery to characterize the size and velocity of the secondary droplets produced by drop impacts onto a heated surface. Exp. Fluids.

[24] Gonzalez R.C., Woods R.E., Eddins S.L. (2004). Digital Image Processing Using Matlab.

[25] Dong X., Zhu H., Yang X. (2013). Three-Dimensional Imaging system for Analyses of Dynamic Droplet Impaction and Deposit Formation on Leaves. Trans. ASABE.

[26] Canny J.A. (1983). Computational approach to edge detection. IEEE Trans. Pattern Anal. Mach. Intell..

[27] Yule A.J. (1978). Large-Scale Structure in the mixing layer of a round jet. J. Fluid Mech..

[28] Lee S., Kim Y. (2004). Sizing of spray particles using image processing technique. KSME Int. J..

[29] Lee C., Wu C.H., Hoopes J.A. (2009). Simultaneous particle size and concentration measurements using a back-lighted particle imaging system. Flow Meas. Instrum..

[30] Huang K.Y., Ye Y.T. (2015). A Novel Machine Vision System on the Inspection of Micro-Spray Nozzle. Sensors.

[31] Baek S.J., Lee S.J. (1996). A new two-frame particle tracking algorithm using match probability. Exp. Fluids.

[32] Hislop E.C. (1987). Can we achieve optimum pesticide deposits?. Asp. Appl. Biol..

[33] Cawood P.N., Robinson T.H., Whittaker S. (1995). An Investigation of Alternative Application Techniques for the Control of Black-Grass. Brighton Crop Prot. Conf. Weeds.

[34] Nuyttens D., De Schampheleire M., Baetens K., Brusselman E., Dekeyser D., Verboven P. (2011). Drift from field crop sprayers using an integrated approach: Results from a five-year study. Trans. ASABE.

[35] Butler Ellis M.C., Tuck C.R., Miller P.C.H. (1997). The effect of some adjuvants on sprays produced by agricultural flat fan nozzles. Crop Prot..

[36] Nuyttens D., Baetens K., De Schampheleire M., Sonck B. (2007). Effect of nozzle type, size and pressure on spray droplet characteristics. Biosyst. Eng..

[37] Dekeyser D., Duga A.T., Verboven P., Hendrickx N., Nuyttens D. (2013). Assessment of orchard sprayers using laboratory experiments and CFD modelling. Biosyst. Eng..

